# The mechanism for stochastic resonance enhancement of mammalian auditory information processing

**DOI:** 10.1186/1742-4682-3-39

**Published:** 2006-12-01

**Authors:** Dawei Hong, Joseph V Martin, William M Saidel

**Affiliations:** 1 Department of Computer Science, Rutgers University, Camden, New Jersey, USA; 2 Department of Biology, Rutgers University, Camden, New Jersey, USA

## Abstract

**Background:**

In a mammalian auditory system, when intrinsic noise is added to a subthreshold signal, not only can the resulting noisy signal be detected, but also the information carried by the signal can be completely recovered. Such a phenomenon is called stochastic resonance (SR). Current analysis of SR commonly employs the energies of the subthreshold signal and intrinsic noise. However, it is difficult to explain SR when the energy addition of the signal and noise is not enough to lift the subthreshold signal over the threshold. Therefore, information modulation has been hypothesized to play a role in some forms of SR in sensory systems. Information modulation, however, seems an unlikely mechanism for mammalian audition, since it requires significant *a priori *knowledge of the characteristics of the signal.

**Results:**

We propose that the analysis of SR cannot rely solely on the energies of a subthreshold signal and intrinsic noise or on information modulation. We note that a mammalian auditory system expends energy in the processing of a noisy signal. A part of the expended energy may therefore deposit into the recovered signal, lifting it over threshold. We propose a model that in a rigorous mathematical manner expresses this new theoretical viewpoint on SR in the mammalian auditory system and provide a physiological rationale for the model.

**Conclusion:**

Our result indicates that the mammalian auditory system may be more active than previously described in the literature. As previously recognized, when intrinsic noise is used to generate a noisy signal, the energy carried by the noise is added to the original subthreshold signal. Furthermore, our model predicts that the system itself should deposit additional energy into the recovered signal. The additional energy is used in the processing of the noisy signal to recover the original subthreshold signal.

## Background

Stochastic resonance (SR) is a phenomenon resulting from the interactions between stochastic processes and many physical systems [1-4]. In the early 1990s, Moss and colleagues [5] pointed out the importance of SR phenomena in biological sensory systems. Subsequently, Moss developed a more general theory (see reviews in [6,7]). We will use the term "SR" for stochastic resonance in biological sensory systems [6]. As a stochastic phenomenon, SR consists of three ingredients: a threshold, a subthreshold signal (the original signal), and intrinsic noise. The original signal is insufficient to reach threshold and stimulate the appropriate sensory system unless it interacts with some intrinsic noise. Such an interaction generates a "noisy signal". When the derived noisy signal exceeds threshold in a sensory system, a sequence of action potentials (the spike train) is produced by the first stages of the system. Subsequent neural processes use these spikes to recover the information contained within the original signal. For a biological sensory system, SR enhances sensory information processing, particularly near the system's threshold.

As summarized in a recently published review [7], a core idea of Moss' theory on SR is that "(t)he role of noise is to sample the stimulus. This means that the larger amplitude excursions of the noise cross the threshold and provide a sample of the subthreshold signal's amplitude at a given instant in time. For good information transmission, the sampling rate should be greater than the stimulus frequency." (p. 269). As noise takes samples (in amplitude) from a subthreshold signal at a series of instants of time, a noisy signal is created. This process can be formulated as follows. An input to the mammalian auditory system, which we will call the original signal, is commonly modeled by a mathematical curve, a function *h*(*t*): *t *∈ [0,1] ↦ ℝ. Here, *h *is supposed, at least, to be continuous; *t *represents time; the time period is normalized as [0,1]; and *h*(*t*) stands for the amplitude of the signal at time instant *t*. The information carried by *h *is encoded in both amplitude and frequency. Noise is commonly modeled by a random variable, which in mathematical terms is a measurable function *e*(*t*): *t *∈ [0,1] ↦ ℝ where *e*(*t*) is the amplitude of the noise at time instant *t*. The noise in a mammalian auditory system is intrinsic. That is, the physiological evidence suggests that noise is generated by the system internally. For the mammalian auditory system, we can set a baseline such that the intensity is zero. Since amplitude is measured by intensity against the baseline, we can let *h*(*t*) > 0 and *e*(*t*) > 0 for all *t *∈ [0,1]. The resulting noisy signal is represented by

*f*(*t*) = *h*(*t*) + *e*(*t*)     (1)

which usually is quite irregular. As previously adopted in the literature on SR in sensory systems [8], (1) indicates that noise is additive with the original signal.

Thus, in the original formulation of Moss' theory [8], the energies of signal and noise were not considered. The theory simply required a mechanism by which addition of the raw data of an original signal *h*(*t*) and noise *e*(*t*) would eventually enhance mammalian auditory information processing.

### Energy Addition and Information Modulation

If the core idea of Moss' theory [8] is valid for mammalian auditory information processing (as we strongly believe), one has to accept that a mammalian auditory system is capable of recovering an original signal *h*(*t*) from the noisy signal *f*(*t*) expressed in (1). Guided by Occam's razor, we expect the mechanism of (1) to be generally applicable in the mammalian auditory system. As a first step, it is natural to analyze the energies carried by the signal *h*(*t*) and noise *e*(*t*). Indeed, in many cases, e.g. [9], the energy addition of the signal and noise is sufficient to explain SR. Moss and his coworkers categorized such SR as Type E (for energy). However, SR has also been observed when the energy addition of the signal and noise is not sufficient to explain the enhancement in sensory perception [10]. Moss *et al*. used the concept of information modulation to explain this observation and categorized such SR as Type I (for information). In other words, the occurrence of of Type I SR relies on characteristics of the signal other than energy. Still, the distinction between Types E and I SR has the disadvantage of requiring evolution of multiple mechanisms for SR in the mammalian auditory system, which would seem less likely than evolution of a single unitary mechanism.

At this point, it is instructional to consider the historical progression of research on signal processing in the latter half of the twentieth century. (We refer the reader to section 1 of [11] for a summary of this history.) Filtering out noise from a noisy signal *f*(*t*) as expressed in (1) is a major concern of the community of signal processing, where this task is termed "de-noising". Early researchers developed a substantial number of algorithms for de-noising. However, most of the de-noising algorithms were mathematically proven to be optimal when the characteristics of original signal *h*(*t*) could be known to the algorithm in advance. De-noising was thought to require information modulation. In 1994, Donoho and Johnstone [12] dramatically changed the modern understanding of de-noising by proposing wavelet shrinkage. Importantly, wavelet-based algorithms do not require *a priori *knowledge of the characteristics of the signal (see below) and can be implemented more efficiently than earlier methods such as the fast Fourier transform (FFT).

### Wavelet Shrinkage

Since our proposed model employs a recent improvement on analysis of wavelet shrinkage, we will mention some details related to this algorithm. Recall that an original signal is modeled by a function *h*(*t*): *t *∈ [0,1] ↦ ℝ^+^. In the mammalian auditory system, *h*(*t*) necessarily has a certain degree of "smoothness". In the literature on signal processing, this is formulated as a requirement that *h*(*t*) belongs to a Hölder class. Recall that a Hölder class Λ^*α*^(*M*) is a family of functions, which is determined by two parameters *α *and *M *as follows: Let ℝ^[0,1] ^denote the set of all functions defined on [0,1]. For 0 <*α *≤ 1, Λ^*α*^(*M*) def¯¯
MathType@MTEF@5@5@+=feaafiart1ev1aaatCvAUfKttLearuWrP9MDH5MBPbIqV92AaeXatLxBI9gBaebbnrfifHhDYfgasaacH8akY=wiFfYdH8Gipec8Eeeu0xXdbba9frFj0=OqFfea0dXdd9vqai=hGuQ8kuc9pgc9s8qqaq=dirpe0xb9q8qiLsFr0=vr0=vr0dc8meaabaqaciaacaGaaeqabaqabeGadaaakeaadaadbaqaaiabbsgaKjabbwgaLjabbAgaMbaaaaa@30B0@ {*h *∈ ℝ^[0,1]^: (∀*x*_1_, *x*_2 _∈ [0,1]), |*h*(*x*_1_) - *h*(*x*_2_)| ≤ *M*|*x*_1 _- *x*_2_|^*α*^}. For 1 <*α*, Λ^*α*^(*M*) def¯¯
MathType@MTEF@5@5@+=feaafiart1ev1aaatCvAUfKttLearuWrP9MDH5MBPbIqV92AaeXatLxBI9gBaebbnrfifHhDYfgasaacH8akY=wiFfYdH8Gipec8Eeeu0xXdbba9frFj0=OqFfea0dXdd9vqai=hGuQ8kuc9pgc9s8qqaq=dirpe0xb9q8qiLsFr0=vr0=vr0dc8meaabaqaciaacaGaaeqabaqabeGadaaakeaadaadbaqaaiabbsgaKjabbwgaLjabbAgaMbaaaaa@30B0@ {*h *∈ ℝ^[0,1]^: (∀*x *∈ [0,1])|*h*'(*x*)| ≤ *M*, *h*^⌊*α*⌋ ^exists, and (∀*x*_1_, *x*_2 _∈ [0,1])|*h*^⌊*α*⌋ ^(*x*_1_) - *h*^⌊*α*⌋ ^(*x*_2_)| ≤ *M*|*x*_1 _- *x*_2_|^*α *- ⌊*α*⌋^}. It is straightforward to see that the concept of Hölder class contains information modulation. For example, a sine wave belongs to a Hölder class with 1 <*α*; however, the higher the frequency of the wave is, the larger the *M *must be. Before the advent of wavelet shrinkage, proposed de-noising algorithms required *α *and *M *as part of their inputs. Unlike the earlier algorithms, wavelet shrinkage only requires that *h*(*t*) belongs to a Hölder class, without further knowledge of *α *and *M*. Therefore, wavelet shrinkage provides a universal solution for de-noising. Strikingly, it was mathematically proven that the recovery of a signal by wavelet shrinkage is as good as that obtained by earlier algorithms requiring specific knowledge of *α *and *M *[12]. Therefore, based on wavelet shrinkage we can propose a model that universally explains SR, including both Types E and I in the same model.

To realize the new model, we must first overcome a mathematical difficulty. Throughout the rest of this paper, we will always denote by h˜
 MathType@MTEF@5@5@+=feaafiart1ev1aaatCvAUfKttLearuWrP9MDH5MBPbIqV92AaeXatLxBI9gBaebbnrfifHhDYfgasaacH8akY=wiFfYdH8Gipec8Eeeu0xXdbba9frFj0=OqFfea0dXdd9vqai=hGuQ8kuc9pgc9s8qqaq=dirpe0xb9q8qiLsFr0=vr0=vr0dc8meaabaqaciaacaGaaeqabaqabeGadaaakeaacuWGObaAgaacaaaa@2E14@(*t*) the signal recovered from a noisy signal as expressed in (1). In signal processing, the performance of a de-noising algorithm is mainly judged by the closeness between the recovered signal h˜
 MathType@MTEF@5@5@+=feaafiart1ev1aaatCvAUfKttLearuWrP9MDH5MBPbIqV92AaeXatLxBI9gBaebbnrfifHhDYfgasaacH8akY=wiFfYdH8Gipec8Eeeu0xXdbba9frFj0=OqFfea0dXdd9vqai=hGuQ8kuc9pgc9s8qqaq=dirpe0xb9q8qiLsFr0=vr0=vr0dc8meaabaqaciaacaGaaeqabaqabeGadaaakeaacuWGObaAgaacaaaa@2E14@(*t*) and original signal *h*(*t*), and this closeness is measured in terms of *L*_2 _norm ||h˜
 MathType@MTEF@5@5@+=feaafiart1ev1aaatCvAUfKttLearuWrP9MDH5MBPbIqV92AaeXatLxBI9gBaebbnrfifHhDYfgasaacH8akY=wiFfYdH8Gipec8Eeeu0xXdbba9frFj0=OqFfea0dXdd9vqai=hGuQ8kuc9pgc9s8qqaq=dirpe0xb9q8qiLsFr0=vr0=vr0dc8meaabaqaciaacaGaaeqabaqabeGadaaakeaacuWGObaAgaacaaaa@2E14@(*t*) - *h*(*t*)||_2 _def¯¯∫01(h˜(t)−h(t))2dt
MathType@MTEF@5@5@+=feaafiart1ev1aaatCvAUfKttLearuWrP9MDH5MBPbIqV92AaeXatLxBI9gBaebbnrfifHhDYfgasaacH8akY=wiFfYdH8Gipec8Eeeu0xXdbba9frFj0=OqFfea0dXdd9vqai=hGuQ8kuc9pgc9s8qqaq=dirpe0xb9q8qiLsFr0=vr0=vr0dc8meaabaqaciaacaGaaeqabaqabeGadaaakeaadaadbaqaaiabbsgaKjabbwgaLjabbAgaMbaadaGcaaqaamaapedabaGaeiikaGIafmiAaGMbaGaacqGGOaakcqWG0baDcqGGPaqkcqGHsislcqWGObaAcqGGOaakcqWG0baDcqGGPaqkcqGGPaqkdaahaaWcbeqaaiabikdaYaaakiabdsgaKjabdsha0bWcbaGaeGimaadabaGaeGymaedaniabgUIiYdaaleqaaaaa@4463@. For SR in the mammalian auditory system, however, when *h*(*t*) has few sharp transients which are lost in h˜
 MathType@MTEF@5@5@+=feaafiart1ev1aaatCvAUfKttLearuWrP9MDH5MBPbIqV92AaeXatLxBI9gBaebbnrfifHhDYfgasaacH8akY=wiFfYdH8Gipec8Eeeu0xXdbba9frFj0=OqFfea0dXdd9vqai=hGuQ8kuc9pgc9s8qqaq=dirpe0xb9q8qiLsFr0=vr0=vr0dc8meaabaqaciaacaGaaeqabaqabeGadaaakeaacuWGObaAgaacaaaa@2E14@, one may still have ||h˜
 MathType@MTEF@5@5@+=feaafiart1ev1aaatCvAUfKttLearuWrP9MDH5MBPbIqV92AaeXatLxBI9gBaebbnrfifHhDYfgasaacH8akY=wiFfYdH8Gipec8Eeeu0xXdbba9frFj0=OqFfea0dXdd9vqai=hGuQ8kuc9pgc9s8qqaq=dirpe0xb9q8qiLsFr0=vr0=vr0dc8meaabaqaciaacaGaaeqabaqabeGadaaakeaacuWGObaAgaacaaaa@2E14@(*t*) - *h*(*t*)||_2 _≈ 0. Of greater concern, for a given original signal *h*(*t*), the recovered signal h˜
 MathType@MTEF@5@5@+=feaafiart1ev1aaatCvAUfKttLearuWrP9MDH5MBPbIqV92AaeXatLxBI9gBaebbnrfifHhDYfgasaacH8akY=wiFfYdH8Gipec8Eeeu0xXdbba9frFj0=OqFfea0dXdd9vqai=hGuQ8kuc9pgc9s8qqaq=dirpe0xb9q8qiLsFr0=vr0=vr0dc8meaabaqaciaacaGaaeqabaqabeGadaaakeaacuWGObaAgaacaaaa@2E14@(*t*) is random. This is because the noise *e*(*t*) is random, and hence, the noisy signal *f*(*t*) = *h*(*t*) + *e*(*t*) is random. While in signal processing, the performance of a de-noising algorithm such as wavelet shrinkage (see [12]), is judged by E [||h˜
 MathType@MTEF@5@5@+=feaafiart1ev1aaatCvAUfKttLearuWrP9MDH5MBPbIqV92AaeXatLxBI9gBaebbnrfifHhDYfgasaacH8akY=wiFfYdH8Gipec8Eeeu0xXdbba9frFj0=OqFfea0dXdd9vqai=hGuQ8kuc9pgc9s8qqaq=dirpe0xb9q8qiLsFr0=vr0=vr0dc8meaabaqaciaacaGaaeqabaqabeGadaaakeaacuWGObaAgaacaaaa@2E14@(*t*) - *h*(*t*)||_2_], the average closeness between h˜
 MathType@MTEF@5@5@+=feaafiart1ev1aaatCvAUfKttLearuWrP9MDH5MBPbIqV92AaeXatLxBI9gBaebbnrfifHhDYfgasaacH8akY=wiFfYdH8Gipec8Eeeu0xXdbba9frFj0=OqFfea0dXdd9vqai=hGuQ8kuc9pgc9s8qqaq=dirpe0xb9q8qiLsFr0=vr0=vr0dc8meaabaqaciaacaGaaeqabaqabeGadaaakeaacuWGObaAgaacaaaa@2E14@(*t*) and *h*(*t*), it would clearly be unacceptable to claim that SR enhances mammalian auditory information processing *on the average*. Fortunately, the performance of wavelet shrinkage can be judged by sup_1≤*t*≤1 _|h˜
 MathType@MTEF@5@5@+=feaafiart1ev1aaatCvAUfKttLearuWrP9MDH5MBPbIqV92AaeXatLxBI9gBaebbnrfifHhDYfgasaacH8akY=wiFfYdH8Gipec8Eeeu0xXdbba9frFj0=OqFfea0dXdd9vqai=hGuQ8kuc9pgc9s8qqaq=dirpe0xb9q8qiLsFr0=vr0=vr0dc8meaabaqaciaacaGaaeqabaqabeGadaaakeaacuWGObaAgaacaaaa@2E14@(*t*) - *h*(*t*)| with very high probability [13]. That is, the signal recovered by wavelet shrinkage is almost surely (with probability 1) close to an original signal, even when examined in a pointwise fashion. In the next section, we will propose a model for SR based on this new result of Hong and Birget [13]. Since in mammalian hearing any part of *h*(*t*) may contain crucial information, a necessary condition to recover an original signal *h*(*t*) from the noisy signal *f*(*t*) = *h*(*t*) + *e*(*t*) is that the noisy signal be detectable. The proposed model will show that in mammalian hearing, SR occurs *if and only if the noisy signal is detectable*. In addition, we will demonstrate that the model explains both so-called Types E and I SR in a unitary mechanism.

In the final section, we will indicate how observed physiological structures and functions in mammalian auditory system are consistent with and suggest the proposed model.

## Results and Discussion

### The proposed model

Recall that in SR the role of noise *e*(*t*) is to sample an original signal *h*(*t*) generating a noisy signal *f*(*t*) = *h*(*t*) + *e*(*t*); and that the sampling rate needs to be greater than the frequency of *h*(*t*) [7]. Mathematically, the sampling of the original signal by noise indicates that SR has a discrete nature. A mammalian auditory system can therefore be viewed as a "device" with the following characteristics. Let *n*, a large positive integer, denote the sampling rate.

**Input**: At time instants *t *= in
 MathType@MTEF@5@5@+=feaafiart1ev1aaatCvAUfKttLearuWrP9MDH5MBPbIqV92AaeXatLxBI9gBaebbnrfifHhDYfgasaacH8akY=wiFfYdH8Gipec8Eeeu0xXdbba9frFj0=OqFfea0dXdd9vqai=hGuQ8kuc9pgc9s8qqaq=dirpe0xb9q8qiLsFr0=vr0=vr0dc8meaabaqaciaacaGaaeqabaqabeGadaaakeaadaWcaaqaaiabdMgaPbqaaiabd6gaUbaaaaa@2F7C@, *i *= 1, 2, ..., *n*, an original subthreshold signal *h*(*t*), *t *∈ [0,1], is sampled by a noise *e*(*t*). This results in the noisy samples *f*(in
 MathType@MTEF@5@5@+=feaafiart1ev1aaatCvAUfKttLearuWrP9MDH5MBPbIqV92AaeXatLxBI9gBaebbnrfifHhDYfgasaacH8akY=wiFfYdH8Gipec8Eeeu0xXdbba9frFj0=OqFfea0dXdd9vqai=hGuQ8kuc9pgc9s8qqaq=dirpe0xb9q8qiLsFr0=vr0=vr0dc8meaabaqaciaacaGaaeqabaqabeGadaaakeaadaWcaaqaaiabdMgaPbqaaiabd6gaUbaaaaa@2F7C@) = *h*(in
 MathType@MTEF@5@5@+=feaafiart1ev1aaatCvAUfKttLearuWrP9MDH5MBPbIqV92AaeXatLxBI9gBaebbnrfifHhDYfgasaacH8akY=wiFfYdH8Gipec8Eeeu0xXdbba9frFj0=OqFfea0dXdd9vqai=hGuQ8kuc9pgc9s8qqaq=dirpe0xb9q8qiLsFr0=vr0=vr0dc8meaabaqaciaacaGaaeqabaqabeGadaaakeaadaWcaaqaaiabdMgaPbqaaiabd6gaUbaaaaa@2F7C@) + *e*(in
 MathType@MTEF@5@5@+=feaafiart1ev1aaatCvAUfKttLearuWrP9MDH5MBPbIqV92AaeXatLxBI9gBaebbnrfifHhDYfgasaacH8akY=wiFfYdH8Gipec8Eeeu0xXdbba9frFj0=OqFfea0dXdd9vqai=hGuQ8kuc9pgc9s8qqaq=dirpe0xb9q8qiLsFr0=vr0=vr0dc8meaabaqaciaacaGaaeqabaqabeGadaaakeaadaWcaaqaaiabdMgaPbqaaiabd6gaUbaaaaa@2F7C@).

**Output**: A recovered signal h˜
 MathType@MTEF@5@5@+=feaafiart1ev1aaatCvAUfKttLearuWrP9MDH5MBPbIqV92AaeXatLxBI9gBaebbnrfifHhDYfgasaacH8akY=wiFfYdH8Gipec8Eeeu0xXdbba9frFj0=OqFfea0dXdd9vqai=hGuQ8kuc9pgc9s8qqaq=dirpe0xb9q8qiLsFr0=vr0=vr0dc8meaabaqaciaacaGaaeqabaqabeGadaaakeaacuWGObaAgaacaaaa@2E14@(*t*) obtained by processing the noisy samples *f*(in
 MathType@MTEF@5@5@+=feaafiart1ev1aaatCvAUfKttLearuWrP9MDH5MBPbIqV92AaeXatLxBI9gBaebbnrfifHhDYfgasaacH8akY=wiFfYdH8Gipec8Eeeu0xXdbba9frFj0=OqFfea0dXdd9vqai=hGuQ8kuc9pgc9s8qqaq=dirpe0xb9q8qiLsFr0=vr0=vr0dc8meaabaqaciaacaGaaeqabaqabeGadaaakeaadaWcaaqaaiabdMgaPbqaaiabd6gaUbaaaaa@2F7C@), *i *= 1, 2, ..., *n*.

Since the noise *e*(*t*) is intrinsic, i.e., generated within the mammalian auditory system, the intensity is clearly always bounded. That is, the random variable *e*(*t*) is bounded. We assume there are two constants 0 ≤ *a *<*b *such that *e*(*t*) ∈ [*a,b*]. The criterion for the closeness between the recovered signal h˜
 MathType@MTEF@5@5@+=feaafiart1ev1aaatCvAUfKttLearuWrP9MDH5MBPbIqV92AaeXatLxBI9gBaebbnrfifHhDYfgasaacH8akY=wiFfYdH8Gipec8Eeeu0xXdbba9frFj0=OqFfea0dXdd9vqai=hGuQ8kuc9pgc9s8qqaq=dirpe0xb9q8qiLsFr0=vr0=vr0dc8meaabaqaciaacaGaaeqabaqabeGadaaakeaacuWGObaAgaacaaaa@2E14@(*t*) and original signal *h*(*t*) is

the smallness ofsup⁡t∈[0,1]|h˜(t)−h(t)|     (2)
 MathType@MTEF@5@5@+=feaafiart1ev1aaatCvAUfKttLearuWrP9MDH5MBPbIqV92AaeXatLxBI9gBaebbnrfifHhDYfgasaacH8akY=wiFfYdH8Gipec8Eeeu0xXdbba9frFj0=OqFfea0dXdd9vqai=hGuQ8kuc9pgc9s8qqaq=dirpe0xb9q8qiLsFr0=vr0=vr0dc8meaabaqaciaacaGaaeqabaqabeGadaaakeaacqqG0baDcqqGObaAcqqGLbqzcqqGGaaicqqGZbWCcqqGTbqBcqqGHbqycqqGSbaBcqqGSbaBcqqGUbGBcqqGLbqzcqqGZbWCcqqGZbWCcqqGGaaicqqGVbWBcqqGMbGzdaWfqaqaaiGbcohaZjabcwha1jabcchaWbWcbaGaemiDaqNaeyicI4Saei4waSLaeGimaaJaeiilaWIaeGymaeJaeiyxa0fabeaakiabcYha8jqbdIgaOzaaiaGaeiikaGIaemiDaqNaeiykaKIaeyOeI0IaemiAaGMaeiikaGIaemiDaqNaeiykaKIaeiiFaWNaaCzcaiaaxMaadaqadaqaaiabikdaYaGaayjkaiaawMcaaaaa@5EDF@

where the meaning of the standard mathematical notation "sup" is as follows. Consider all upper bounds for_P_(·) where (·) stands for an expression and P is a predicate which the expression must satisfy. Then, sup_P_(·) is the smallest possible of all the upper bounds.

The procedure to process the noisy samples follows from the notion of wavelet shrinkage in signal processing. It consists of two linear transforms and one non-linear thresholding. That is, we model a mammalian auditory system as a non-linear system.

First, a linear transform is carried out to decompose the noisy samples in the cochlea. For simplicity, in accordance with (1) we denote the noisy samples by *f*. Auditory information carried by the original signal *h*(*t*) is encoded by the changes in both amplitude and frequency. Hence, retrieval of the information from *h*(*t*) requires its decomposition according to both amplitude and frequency. Now, *h*(*t*) is mixed with a noise *e*(*t*), generating *f*(*t*); and the function of the auditory system is to process the noisy samples *f*. Thus, a decomposition of *f *is necessary at the very beginning of the procedure. The principle for such a decomposition is as follows: *f *is viewed as an element in a function space, usually *L*^2^[0,1]; and then, with the choice of a basis of *L*^2^[0,1], it finds the projections of *f *on each component in the basis. Thus, the mathematical quality of the decomposition is determined by the basis. Technically, during mammalian auditory information processing, the noisy samples *f *are decomposed to allow recovery of *h*(*t*). Any basis that is chosen for the decomposition must be sensitive in detecting changes in both amplitude and frequency at the same time. It is mathematically proven that a wavelet basis is the best choice for such a decomposition. While there are many wavelet bases, from the Haar to the Daubechies, we do not specify a particular wavelet basis in the proposed model, except that it is required to be orthonormal.

It must be noted that once a wavelet basis is chosen, the linear transform is constant in the following sense. Recall that in a standard way, a linear transform can be represented by a matrix and vice versa. The matrix representing this linear transform is constant if all entries in the matrix are constants. From a viewpoint of physiology, this indicates that once a mammalian auditory system is developed, it may decompose signals to filter out noise in a fixed manner.

Since the first linear transform decomposes the noisy samples, it is necessary to filter out the noise right after this transform. A non-linear thresholding is applied immediately as the second step in the procedure. It also must be noted that the threshold here is again a constant if the sampling rate *n *is regarded as fixed. The output from the second step is the decomposed noisy samples with the noise filtered out. Thus, the third (final) step of the procedure is to re-compose the filtered output of the second step. It is carried out by a linear transform, which again is constant in the sense mentioned above for the first step.

Mathematically, we describe the three steps as follows. Two related *n *× *n *orthonormal matrices *V *and V˜
 MathType@MTEF@5@5@+=feaafiart1ev1aaatCvAUfKttLearuWrP9MDH5MBPbIqV92AaeXatLxBI9gBaebbnrfifHhDYfgasaacH8akY=wiFfYdH8Gipec8Eeeu0xXdbba9frFj0=OqFfea0dXdd9vqai=hGuQ8kuc9pgc9s8qqaq=dirpe0xb9q8qiLsFr0=vr0=vr0dc8meaabaqaciaacaGaaeqabaqabeGadaaakeaacuWGwbGvgaacaaaa@2DF0@ respectively for a discrete wavelet transform (DWT) and its inverse are used for the first and third steps, respectively.

V=(v11v12…v1nv21v22…v2n…vn1vn2…vnn)
 MathType@MTEF@5@5@+=feaafiart1ev1aaatCvAUfKttLearuWrP9MDH5MBPbIqV92AaeXatLxBI9gBaebbnrfifHhDYfgasaacH8akY=wiFfYdH8Gipec8Eeeu0xXdbba9frFj0=OqFfea0dXdd9vqai=hGuQ8kuc9pgc9s8qqaq=dirpe0xb9q8qiLsFr0=vr0=vr0dc8meaabaqaciaacaGaaeqabaqabeGadaaakeaacqWGwbGvcqGH9aqpdaqadaqaauaabmqaeqaaaaaabaGaemODay3aaSbaaSqaaiabigdaXiabigdaXaqabaaakeaacqWG2bGDdaWgaaWcbaGaeGymaeJaeGOmaidabeaaaOqaaiablAcilbqaaiabdAha2naaBaaaleaacqaIXaqmcqWGUbGBaeqaaaGcbaGaemODay3aaSbaaSqaaiabikdaYiabigdaXaqabaaakeaacqWG2bGDdaWgaaWcbaGaeGOmaiJaeGOmaidabeaaaOqaaiablAcilbqaaiabdAha2naaBaaaleaacqaIYaGmcqWGUbGBaeqaaaGcbaaabaaabaGaeSOjGSeabaaabaGaemODay3aaSbaaSqaaiabd6gaUjabigdaXaqabaaakeaacqWG2bGDdaWgaaWcbaGaemOBa4MaeGOmaidabeaaaOqaaiablAcilbqaaiabdAha2naaBaaaleaacqWGUbGBcqWGUbGBaeqaaaaaaOGaayjkaiaawMcaaaaa@57C7@

and

V˜=(v11˜v12˜…v1n˜v21˜v22˜…v2n˜…vn1˜vn2˜…vnn˜)
 MathType@MTEF@5@5@+=feaafiart1ev1aaatCvAUfKttLearuWrP9MDH5MBPbIqV92AaeXatLxBI9gBaebbnrfifHhDYfgasaacH8akY=wiFfYdH8Gipec8Eeeu0xXdbba9frFj0=OqFfea0dXdd9vqai=hGuQ8kuc9pgc9s8qqaq=dirpe0xb9q8qiLsFr0=vr0=vr0dc8meaabaqaciaacaGaaeqabaqabeGadaaakeaacuWGwbGvgaacaiabg2da9maabmaabaqbaeWabqabaaaaaeaadaaiaaqaaiabdAha2naaBaaaleaacqaIXaqmcqaIXaqmaeqaaaGccaGLdmaaaeaadaaiaaqaaiabdAha2naaBaaaleaacqaIXaqmcqaIYaGmaeqaaaGccaGLdmaaaeaacqWIMaYsaeaadaaiaaqaaiabdAha2naaBaaaleaacqaIXaqmcqWGUbGBaeqaaaGccaGLdmaaaeaadaaiaaqaaiabdAha2naaBaaaleaacqaIYaGmcqaIXaqmaeqaaaGccaGLdmaaaeaadaaiaaqaaiabdAha2naaBaaaleaacqaIYaGmcqaIYaGmaeqaaaGccaGLdmaaaeaacqWIMaYsaeaadaaiaaqaaiabdAha2naaBaaaleaacqaIYaGmcqWGUbGBaeqaaaGccaGLdmaaaeaaaeaaaeaacqWIMaYsaeaaaeaadaaiaaqaaiabdAha2naaBaaaleaacqWGUbGBcqaIXaqmaeqaaaGccaGLdmaaaeaadaaiaaqaaiabdAha2naaBaaaleaacqWGUbGBcqaIYaGmaeqaaaGccaGLdmaaaeaacqWIMaYsaeaadaaiaaqaaiabdAha2naaBaaaleaacqWGUbGBcqWGUbGBaeqaaaGccaGLdmaaaaaacaGLOaGaayzkaaaaaa@5EA8@

For a mammalian auditory system, the two matrices are fixed, i.e. *v*_*ij *_and vij˜
 MathType@MTEF@5@5@+=feaafiart1ev1aaatCvAUfKttLearuWrP9MDH5MBPbIqV92AaeXatLxBI9gBaebbnrfifHhDYfgasaacH8akY=wiFfYdH8Gipec8Eeeu0xXdbba9frFj0=OqFfea0dXdd9vqai=hGuQ8kuc9pgc9s8qqaq=dirpe0xb9q8qiLsFr0=vr0=vr0dc8meaabaqaciaacaGaaeqabaqabeGadaaakeaadaaiaaqaaiabdAha2naaBaaaleaacqWGPbqAcqWGQbGAaeqaaaGccaGLdmaaaaa@31D1@ are fixed during development, and they are used to process any noisy signal entering the system.

A threshold for the second step is defined as

λn,δ=c⋅(b−a)⋅(1+21+δ)ln⁡2)log⁡2nn
 MathType@MTEF@5@5@+=feaafiart1ev1aaatCvAUfKttLearuWrP9MDH5MBPbIqV92AaeXatLxBI9gBaebbnrfifHhDYfgasaacH8akY=wiFfYdH8Gipec8Eeeu0xXdbba9frFj0=OqFfea0dXdd9vqai=hGuQ8kuc9pgc9s8qqaq=dirpe0xb9q8qiLsFr0=vr0=vr0dc8meaabaqaciaacaGaaeqabaqabeGadaaakeaaiiGacqWF7oaBdaWgaaWcbaGaemOBa4MaeiilaWIae8hTdqgabeaakiabg2da9iabdogaJjabgwSixlabcIcaOiabdkgaIjabgkHiTiabdggaHjabcMcaPiabgwSixpaabmaabaGaeGymaeJaey4kaSIaeGOmaiZaaOaaaeaacqaIXaqmcqGHRaWkcqWF0oazcqGGPaqkcyGGSbaBcqGGUbGBcqaIYaGmaSqabaaakiaawIcacaGLPaaadaGcaaqaamaalaaabaGagiiBaWMaei4Ba8Maei4zaC2aaSbaaSqaaiabikdaYaqabaGccqWGUbGBaeaacqWGUbGBaaaaleqaaaaa@5353@

where *c *> 0 is a parameter determined according to which wavelet basis is used, and *δ *> 0 is a parameter related to the accuracy of the auditory information processing. The threshold *λ*_*n,δ *_is different from the threshold *s *used by the spike train. Notice that for an auditory system, *λ*_*n,δ *_is fixed (recall that [*a, b*] is the range of the intrinsic noise) and is used to process any noisy signal entering the auditory system.

In what follows, we will use some simplified notations. We let *h*_*i *_denote *h*(in
 MathType@MTEF@5@5@+=feaafiart1ev1aaatCvAUfKttLearuWrP9MDH5MBPbIqV92AaeXatLxBI9gBaebbnrfifHhDYfgasaacH8akY=wiFfYdH8Gipec8Eeeu0xXdbba9frFj0=OqFfea0dXdd9vqai=hGuQ8kuc9pgc9s8qqaq=dirpe0xb9q8qiLsFr0=vr0=vr0dc8meaabaqaciaacaGaaeqabaqabeGadaaakeaadaWcaaqaaiabdMgaPbqaaiabd6gaUbaaaaa@2F7C@), *i *= 1,2, ..., *n*; and let *h *= (*h*_1 _*h*_2 _... *h*_*n*_)^*T *^where (·)^T ^stands for the transposition of a vector (·). We apply the same notation to *e*_*i*_, *f*_*i*_, and h˜
 MathType@MTEF@5@5@+=feaafiart1ev1aaatCvAUfKttLearuWrP9MDH5MBPbIqV92AaeXatLxBI9gBaebbnrfifHhDYfgasaacH8akY=wiFfYdH8Gipec8Eeeu0xXdbba9frFj0=OqFfea0dXdd9vqai=hGuQ8kuc9pgc9s8qqaq=dirpe0xb9q8qiLsFr0=vr0=vr0dc8meaabaqaciaacaGaaeqabaqabeGadaaakeaacuWGObaAgaacaaaa@2E14@_*i*_. Now, we mathematically formulate the three steps mentioned above.

**Step 1 **Discrete wavelet transform (DWT)

n(η1η2⋮ηn)⇐(v11v12…v1nv21v22…v2n…vn1vn2…vnn)(h1h2⋮hn)
 MathType@MTEF@5@5@+=feaafiart1ev1aaatCvAUfKttLearuWrP9MDH5MBPbIqV92AaeXatLxBI9gBaebbnrfifHhDYfgasaacH8akY=wiFfYdH8Gipec8Eeeu0xXdbba9frFj0=OqFfea0dXdd9vqai=hGuQ8kuc9pgc9s8qqaq=dirpe0xb9q8qiLsFr0=vr0=vr0dc8meaabaqaciaacaGaaeqabaqabeGadaaakeaadaGcaaqaaiabd6gaUbWcbeaakmaabmaabaqbaeqabqqaaaaabaacciGae83TdG2aaSbaaSqaaiabigdaXaqabaaakeaacqWF3oaAdaWgaaWcbaGaeGOmaidabeaaaOqaaiabl6Uinbqaaiab=D7aOnaaBaaaleaacqWGUbGBaeqaaaaaaOGaayjkaiaawMcaaiabgcDiCpaabmaabaqbaeWabqabaaaaaeaacqWG2bGDdaWgaaWcbaGaeGymaeJaeGymaedabeaaaOqaaiabdAha2naaBaaaleaacqaIXaqmcqaIYaGmaeqaaaGcbaGaeSOjGSeabaGaemODay3aaSbaaSqaaiabigdaXiabd6gaUbqabaaakeaacqWG2bGDdaWgaaWcbaGaeGOmaiJaeGymaedabeaaaOqaaiabdAha2naaBaaaleaacqaIYaGmcqaIYaGmaeqaaaGcbaGaeSOjGSeabaGaemODay3aaSbaaSqaaiabikdaYiabd6gaUbqabaaakeaaaeaaaeaacqWIMaYsaeaaaeaacqWG2bGDdaWgaaWcbaGaemOBa4MaeGymaedabeaaaOqaaiabdAha2naaBaaaleaacqWGUbGBcqaIYaGmaeqaaaGcbaGaeSOjGSeabaGaemODay3aaSbaaSqaaiabd6gaUjabd6gaUbqabaaaaaGccaGLOaGaayzkaaWaaeWaaeaafaqabeabbaaaaeaacqWGObaAdaWgaaWcbaGaeGymaedabeaaaOqaaiabdIgaOnaaBaaaleaacqaIYaGmaeqaaaGcbaGaeSO7I0eabaGaemiAaG2aaSbaaSqaaiabd6gaUbqabaaaaaGccaGLOaGaayzkaaaaaa@715D@

where (*η*_1 _*η*_2 _... *η*_n_)^T ^is the decomposed noisy samples.

**Step 2 **Thresholding

(ζ1ζ2⋮ζn)⇐(η1η2⋮ηn)
 MathType@MTEF@5@5@+=feaafiart1ev1aaatCvAUfKttLearuWrP9MDH5MBPbIqV92AaeXatLxBI9gBaebbnrfifHhDYfgasaacH8akY=wiFfYdH8Gipec8Eeeu0xXdbba9frFj0=OqFfea0dXdd9vqai=hGuQ8kuc9pgc9s8qqaq=dirpe0xb9q8qiLsFr0=vr0=vr0dc8meaabaqaciaacaGaaeqabaqabeGadaaakeaadaqadaqaauaabeqaeeaaaaqaaGGaciab=z7a6naaBaaaleaacqaIXaqmaeqaaaGcbaGae8NTdO3aaSbaaSqaaiabikdaYaqabaaakeaacqWIUlstaeaacqWF2oGEdaWgaaWcbaGaemOBa4gabeaaaaaakiaawIcacaGLPaaacqGHqhc3daqadaqaauaabeqaeeaaaaqaaiab=D7aOnaaBaaaleaacqaIXaqmaeqaaaGcbaGae83TdG2aaSbaaSqaaiabikdaYaqabaaakeaacqWIUlstaeaacqWF3oaAdaWgaaWcbaGaemOBa4gabeaaaaaakiaawIcacaGLPaaaaaa@4810@

where (*ζ*_1 _*ζ*_2 _... *ζ*_*n*_)^T ^is the result of filtering out the noise from the decomposed noisy samples, which is obtained by

ζi={ηiif|ηi|≥λn,δ0otherwisefor i=1,...,n
 MathType@MTEF@5@5@+=feaafiart1ev1aaatCvAUfKttLearuWrP9MDH5MBPbIqV92AaeXatLxBI9gBaebbnrfifHhDYfgasaacH8akY=wiFfYdH8Gipec8Eeeu0xXdbba9frFj0=OqFfea0dXdd9vqai=hGuQ8kuc9pgc9s8qqaq=dirpe0xb9q8qiLsFr0=vr0=vr0dc8meaabaqaciaacaGaaeqabaqabeGadaaakeaaiiGacqWF2oGEdaWgaaWcbaGaemyAaKgabeaakiabg2da9maaceqabaqbaeqabeGaaaqaauaabaqaciaaaeaacqWF3oaAdaWgaaWcbaGaemyAaKgabeaaaOqaaGqaaiab+LgaPjab+zgaMjabcYha8jab=D7aOnaaBaaaleaacqWGPbqAaeqaaOGaeiiFaWNaeyyzImRae83UdW2aaSbaaSqaaiabd6gaUjabcYcaSiab=r7aKbqabaaakeaacqaIWaamaeaacqqGVbWBcqqG0baDcqqGObaAcqqGLbqzcqqGYbGCcqqG3bWDcqqGPbqAcqqGZbWCcqqGLbqzaaaabaGaeeOzayMaee4Ba8MaeeOCaiNaeeiiaaIaemyAaKMaeyypa0JaeGymaeJaeiilaWIaeiOla4IaeiOla4IaeiOla4IaeiilaWIaemOBa4gaaaGaay5Eaaaaaa@615A@

**Step 3 **Inverse of DWT

(h˜1h˜2⋮h˜n)⇐(v11˜v12˜…v1n˜v21˜v22˜…v2n˜…vn1˜vn2˜…vnn˜)(ζ1ζ2⋮ζn)
 MathType@MTEF@5@5@+=feaafiart1ev1aaatCvAUfKttLearuWrP9MDH5MBPbIqV92AaeXatLxBI9gBaebbnrfifHhDYfgasaacH8akY=wiFfYdH8Gipec8Eeeu0xXdbba9frFj0=OqFfea0dXdd9vqai=hGuQ8kuc9pgc9s8qqaq=dirpe0xb9q8qiLsFr0=vr0=vr0dc8meaabaqaciaacaGaaeqabaqabeGadaaakeaadaqadaqaauaabeqaeeaaaaqaaiqbdIgaOzaaiaWaaSbaaSqaaiabigdaXaqabaaakeaacuWGObaAgaacamaaBaaaleaacqaIYaGmaeqaaaGcbaGaeSO7I0eabaGafmiAaGMbaGaadaWgaaWcbaGaemOBa4gabeaaaaaakiaawIcacaGLPaaacqGHqhc3daqadaqaauaabmqaeqaaaaaabaWaaacaaeaacqWG2bGDdaWgaaWcbaGaeGymaeJaeGymaedabeaaaOGaay5adaaabaWaaacaaeaacqWG2bGDdaWgaaWcbaGaeGymaeJaeGOmaidabeaaaOGaay5adaaabaGaeSOjGSeabaWaaacaaeaacqWG2bGDdaWgaaWcbaGaeGymaeJaemOBa4gabeaaaOGaay5adaaabaWaaacaaeaacqWG2bGDdaWgaaWcbaGaeGOmaiJaeGymaedabeaaaOGaay5adaaabaWaaacaaeaacqWG2bGDdaWgaaWcbaGaeGOmaiJaeGOmaidabeaaaOGaay5adaaabaGaeSOjGSeabaWaaacaaeaacqWG2bGDdaWgaaWcbaGaeGOmaiJaemOBa4gabeaaaOGaay5adaaabaaabaaabaGaeSOjGSeabaaabaWaaacaaeaacqWG2bGDdaWgaaWcbaGaemOBa4MaeGymaedabeaaaOGaay5adaaabaWaaacaaeaacqWG2bGDdaWgaaWcbaGaemOBa4MaeGOmaidabeaaaOGaay5adaaabaGaeSOjGSeabaWaaacaaeaacqWG2bGDdaWgaaWcbaGaemOBa4MaemOBa4gabeaaaOGaay5adaaaaaGaayjkaiaawMcaamaabmaabaqbaeqabqqaaaaabaacciGae8NTdO3aaSbaaSqaaiabigdaXaqabaaakeaacqWF2oGEdaWgaaWcbaGaeGOmaidabeaaaOqaaiabl6Uinbqaaiab=z7a6naaBaaaleaacqWGUbGBaeqaaaaaaOGaayjkaiaawMcaaaaa@7705@

We refer the reader to chapters 4 and 6 of [14] for details on DWT, thresholding, and the inverse of DWT. This work also describes the nature of simple constant matrices *V *and V˜
 MathType@MTEF@5@5@+=feaafiart1ev1aaatCvAUfKttLearuWrP9MDH5MBPbIqV92AaeXatLxBI9gBaebbnrfifHhDYfgasaacH8akY=wiFfYdH8Gipec8Eeeu0xXdbba9frFj0=OqFfea0dXdd9vqai=hGuQ8kuc9pgc9s8qqaq=dirpe0xb9q8qiLsFr0=vr0=vr0dc8meaabaqaciaacaGaaeqabaqabeGadaaakeaacuWGwbGvgaacaaaa@2DF0@, once a wavelet basis is chosen and the sampling *n *is given. Importantly, the three steps can be carried out within time of an order of *n*, i.e., the amount of time needed to process the noisy samples is only proportional to the number *n *of noisy samples. As the sampling rate *n *can be thought as fixed in a mammalian auditory system, the three steps can process an auditory signal as a stream. In other words, as a linear function of *n*, the processing rate of the proposed model is as rapid as conceivably possible. In contrast, the processing rate of a FFT is *n *× log *n*, so that the delay in processing with an increase in *n *would preclude online processing.

### Mathematical analysis

We set a baseline for the mammalian auditory system. In terms of hearing, this baseline is understood as "absolute silence". Mathematically, the baseline is represented by a constant 0. All signals, including noise, are measured against this baseline and are evaluated in terms of pressure. In this way, *h*(*t*), *e*(*t*), *f*(*t*) and h˜
 MathType@MTEF@5@5@+=feaafiart1ev1aaatCvAUfKttLearuWrP9MDH5MBPbIqV92AaeXatLxBI9gBaebbnrfifHhDYfgasaacH8akY=wiFfYdH8Gipec8Eeeu0xXdbba9frFj0=OqFfea0dXdd9vqai=hGuQ8kuc9pgc9s8qqaq=dirpe0xb9q8qiLsFr0=vr0=vr0dc8meaabaqaciaacaGaaeqabaqabeGadaaakeaacuWGObaAgaacaaaa@2E14@(*t*) take positive values. Setting up such a baseline serves the following two purposes:

1. The baseline is fixed, yielding a metric system. For any given mammalian auditory system, our analysis of SR depends on this fixed metric system.

2. While our analysis of SR is *not *concerned with energy, we will be able to compute energy based on the metric system, so as to to show how the proposed model naturally covers all types of SR in the mammalian auditory system.

**Figure 1:**
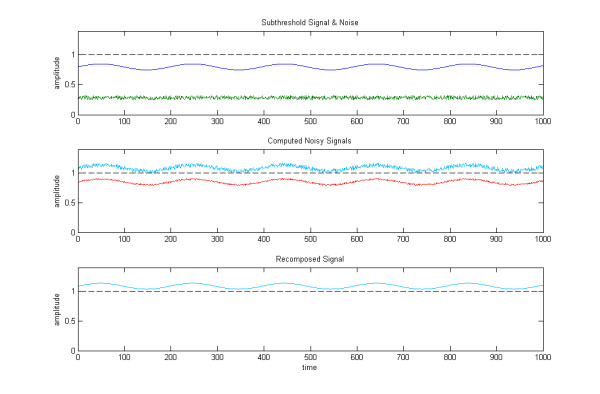
**Comparison of energy addition and geometric translation of a signal by noise**. In each panel the amplitude is plotted as a function of time and threshold is indicated by a horizontal dotted line. Top Panel: Intrinsic noise *ε *is plotted on the lowest (green) trace. The sinusoidal wave represents the subthreshold signal *h *(dark blue). Middle panel: The traces indicate the interaction of *h *and noise to obtain a noisy signal by energy addition(red) or by geometric translation (light blue). The noisy signal obtained by energy addition (red) does not reach threshold, so the SR would traditionally be classified as Type I. However, if the original subthreshold signal *h *is translated by the intrinsic noise *ε *with its mean *m*, the noisy signal obtained by geometric translation (light blue) is entirely above threshold. Lower Panel: As a result of Steps 1,2 and 3, the "denoised" signal is recovered entirely above threshold.

We can assume that the threshold for a stimulus to be detected by the system is *s *> 0. Here, *s *is a constant against the baseline, i.e., the threshold is fixed (see Figure 1). Recall that within time interval [0,1], noise *e*(*t*) samples an original subthreshold signal *h*(*t*), generating the noisy samples *f*_*i *_= *h*_*i *_+ *e*_*i *_where *f*_*i *_= *f*(in
 MathType@MTEF@5@5@+=feaafiart1ev1aaatCvAUfKttLearuWrP9MDH5MBPbIqV92AaeXatLxBI9gBaebbnrfifHhDYfgasaacH8akY=wiFfYdH8Gipec8Eeeu0xXdbba9frFj0=OqFfea0dXdd9vqai=hGuQ8kuc9pgc9s8qqaq=dirpe0xb9q8qiLsFr0=vr0=vr0dc8meaabaqaciaacaGaaeqabaqabeGadaaakeaadaWcaaqaaiabdMgaPbqaaiabd6gaUbaaaaa@2F7C@), *h*_*i *_= *h*(in
 MathType@MTEF@5@5@+=feaafiart1ev1aaatCvAUfKttLearuWrP9MDH5MBPbIqV92AaeXatLxBI9gBaebbnrfifHhDYfgasaacH8akY=wiFfYdH8Gipec8Eeeu0xXdbba9frFj0=OqFfea0dXdd9vqai=hGuQ8kuc9pgc9s8qqaq=dirpe0xb9q8qiLsFr0=vr0=vr0dc8meaabaqaciaacaGaaeqabaqabeGadaaakeaadaWcaaqaaiabdMgaPbqaaiabd6gaUbaaaaa@2F7C@), *e*_*i *_= *e*(in
 MathType@MTEF@5@5@+=feaafiart1ev1aaatCvAUfKttLearuWrP9MDH5MBPbIqV92AaeXatLxBI9gBaebbnrfifHhDYfgasaacH8akY=wiFfYdH8Gipec8Eeeu0xXdbba9frFj0=OqFfea0dXdd9vqai=hGuQ8kuc9pgc9s8qqaq=dirpe0xb9q8qiLsFr0=vr0=vr0dc8meaabaqaciaacaGaaeqabaqabeGadaaakeaadaWcaaqaaiabdMgaPbqaaiabd6gaUbaaaaa@2F7C@), *i *= 1, 2, ..., *n*. Since in mammalian auditory information processing, any *h*_*i *_may contain a critical part of the information carried by *h*, a necessary condition for SR to occur is

*f*_*i *_= *h*_*i *_+ *e*_*i *_≥ *s *for all 1 ≤ *i *≤ *n *    (3)

That is, all noisy samples must be detectable. However, the detectability of the samples does not at all automatically imply that the information carried by *h*(*t*) is retrievable, since at the moment of acquisition, the samples are a mixture of signal *h*(*t*) and noise *e*(*t*).

Our goal is to use the proposed model to prove that the necessary condition expressed in (3) is also a sufficient condition for SR to occur. In precise mathematical terms, if all the noisy samples are detectable, then the information carried by *h*(*t*) can be retrieved almost surely.

Recall that an original signal is represented by a function *h*(*t*): *t *∈ [0,1] ↦ ℝ^+ ^in a Hölder class Λ^*α*^(*M*). It is straightforward to see that for all *t*_1_, *t*_2 _∈ [0,1]

|*h*(*t*_1_) - *h*(*t*_2_)| ≤ *M*|*t*_1 _- *t*_2_|^*α*^

which implies that for all 1 ≤ *i *≤ *n *and for all t∈[i−1n,in]
 MathType@MTEF@5@5@+=feaafiart1ev1aaatCvAUfKttLearuWrP9MDH5MBPbIqV92AaeXatLxBI9gBaebbnrfifHhDYfgasaacH8akY=wiFfYdH8Gipec8Eeeu0xXdbba9frFj0=OqFfea0dXdd9vqai=hGuQ8kuc9pgc9s8qqaq=dirpe0xb9q8qiLsFr0=vr0=vr0dc8meaabaqaciaacaGaaeqabaqabeGadaaakeaacqWG0baDcqGHiiIZdaWadaqaamaalaaabaGaemyAaKMaeyOeI0IaeGymaedabaGaemOBa4gaaiabcYcaSmaalaaabaGaemyAaKgabaGaemOBa4gaaaGaay5waiaaw2faaaaa@39F0@

|h(t)−hi|≤M(1n)α     (4)
 MathType@MTEF@5@5@+=feaafiart1ev1aaatCvAUfKttLearuWrP9MDH5MBPbIqV92AaeXatLxBI9gBaebbnrfifHhDYfgasaacH8akY=wiFfYdH8Gipec8Eeeu0xXdbba9frFj0=OqFfea0dXdd9vqai=hGuQ8kuc9pgc9s8qqaq=dirpe0xb9q8qiLsFr0=vr0=vr0dc8meaabaqaciaacaGaaeqabaqabeGadaaakeaacqGG8baFcqWGObaAcqGGOaakcqWG0baDcqGGPaqkcqGHsislcqWGObaAdaWgaaWcbaGaemyAaKgabeaakiabcYha8jabgsMiJkabd2eannaabmaabaWaaSaaaeaacqaIXaqmaeaacqWGUbGBaaaacaGLOaGaayzkaaWaaWbaaSqabeaaiiGacqWFXoqyaaGccaWLjaGaaCzcamaabmaabaGaeGinaqdacaGLOaGaayzkaaaaaa@4465@

In physiological terms, (4) indicates that a mammalian auditory system uses its intrinsic noise to sample the original signal at a high rate so that information loss between two consecutive samples is negligible. On the other hand, (4) implies that the capability of a mammalian auditory system is limited; it has to lose some information between two consecutive noisy samples. Mathematically, with (4) we need to focus only on h˜
 MathType@MTEF@5@5@+=feaafiart1ev1aaatCvAUfKttLearuWrP9MDH5MBPbIqV92AaeXatLxBI9gBaebbnrfifHhDYfgasaacH8akY=wiFfYdH8Gipec8Eeeu0xXdbba9frFj0=OqFfea0dXdd9vqai=hGuQ8kuc9pgc9s8qqaq=dirpe0xb9q8qiLsFr0=vr0=vr0dc8meaabaqaciaacaGaaeqabaqabeGadaaakeaacuWGObaAgaacaaaa@2E14@ = (h˜
 MathType@MTEF@5@5@+=feaafiart1ev1aaatCvAUfKttLearuWrP9MDH5MBPbIqV92AaeXatLxBI9gBaebbnrfifHhDYfgasaacH8akY=wiFfYdH8Gipec8Eeeu0xXdbba9frFj0=OqFfea0dXdd9vqai=hGuQ8kuc9pgc9s8qqaq=dirpe0xb9q8qiLsFr0=vr0=vr0dc8meaabaqaciaacaGaaeqabaqabeGadaaakeaacuWGObaAgaacaaaa@2E14@_1 _h˜
 MathType@MTEF@5@5@+=feaafiart1ev1aaatCvAUfKttLearuWrP9MDH5MBPbIqV92AaeXatLxBI9gBaebbnrfifHhDYfgasaacH8akY=wiFfYdH8Gipec8Eeeu0xXdbba9frFj0=OqFfea0dXdd9vqai=hGuQ8kuc9pgc9s8qqaq=dirpe0xb9q8qiLsFr0=vr0=vr0dc8meaabaqaciaacaGaaeqabaqabeGadaaakeaacuWGObaAgaacaaaa@2E14@_2 _... h˜
 MathType@MTEF@5@5@+=feaafiart1ev1aaatCvAUfKttLearuWrP9MDH5MBPbIqV92AaeXatLxBI9gBaebbnrfifHhDYfgasaacH8akY=wiFfYdH8Gipec8Eeeu0xXdbba9frFj0=OqFfea0dXdd9vqai=hGuQ8kuc9pgc9s8qqaq=dirpe0xb9q8qiLsFr0=vr0=vr0dc8meaabaqaciaacaGaaeqabaqabeGadaaakeaacuWGObaAgaacaaaa@2E14@_*n*_), the recovery from the noisy samples obtained in Step 3.

In signal processing, the criterion to judge the quality of the recovery h˜
 MathType@MTEF@5@5@+=feaafiart1ev1aaatCvAUfKttLearuWrP9MDH5MBPbIqV92AaeXatLxBI9gBaebbnrfifHhDYfgasaacH8akY=wiFfYdH8Gipec8Eeeu0xXdbba9frFj0=OqFfea0dXdd9vqai=hGuQ8kuc9pgc9s8qqaq=dirpe0xb9q8qiLsFr0=vr0=vr0dc8meaabaqaciaacaGaaeqabaqabeGadaaakeaacuWGObaAgaacaaaa@2E14@ is the average squared error E[Qavg(n)] def¯¯ E[1n∑i=1n(hi−h˜i)2]
MathType@MTEF@5@5@+=feaafiart1ev1aaatCvAUfKttLearuWrP9MDH5MBPbIqV92AaeXatLxBI9gBaebbnrfifHhDYfgasaacH8akY=wiFfYdH8Gipec8Eeeu0xXdbba9frFj0=OqFfea0dXdd9vqai=hGuQ8kuc9pgc9s8qqaq=dirpe0xb9q8qiLsFr0=vr0=vr0dc8meaabaqaciaacaGaaeqabaqabeGadaaakeaacqqGfbqrcqGGBbWwcqWGrbqudaWgaaWcbaGaeeyyaeMaeeODayNaee4zaCgabeaakiabcIcaOiabd6gaUjabcMcaPiabc2faDjabbccaGmaameaabaGaeeizaqMaeeyzauMaeeOzaygaaiabbccaGiabbweafnaadmaabaWaaSaaaeaacqaIXaqmaeaacqWGUbGBaaWaaabmaeaacqGGOaakcqWGObaAdaWgaaWcbaGaemyAaKgabeaakiabgkHiTiqbdIgaOzaaiaWaaSbaaSqaaiabdMgaPbqabaGccqGGPaqkdaahaaWcbeqaaiabikdaYaaaaeaacqWGPbqAcqGH9aqpcqaIXaqmaeaacqWGUbGBa0GaeyyeIuoaaOGaay5waiaaw2faaaaa@5418@, yet, as discussed in the previous section, this criterion is not acceptable for mammalian auditory information processing.

It was mathematically proven that for a Hölder class Λ^*α*^(*M*) the best that any de-noising algorithm can achieve is

E[Qavg(n)]≤C⋅(log⁡2nn)2α1+2α     (5)
 MathType@MTEF@5@5@+=feaafiart1ev1aaatCvAUfKttLearuWrP9MDH5MBPbIqV92AaeXatLxBI9gBaebbnrfifHhDYfgasaacH8akY=wiFfYdH8Gipec8Eeeu0xXdbba9frFj0=OqFfea0dXdd9vqai=hGuQ8kuc9pgc9s8qqaq=dirpe0xb9q8qiLsFr0=vr0=vr0dc8meaabaqaciaacaGaaeqabaqabeGadaaakeaaieaacqWFfbqrcqGGBbWwcqWGrbqudaWgaaWcbaGaeeyyaeMaeeODayNaee4zaCgabeaakiabcIcaOiabd6gaUjabcMcaPiabc2faDjabgsMiJkabdoeadjabgwSixpaabmaabaWaaSaaaeaacyGGSbaBcqGGVbWBcqGGNbWzdaWgaaWcbaGaeGOmaidabeaakiabd6gaUbqaaiabd6gaUbaaaiaawIcacaGLPaaadaahaaWcbeqaamaalaaabaGaeGOmaidcciGae4xSdegabaGaeGymaeJaey4kaSIaeGOmaiJae4xSdegaaaaakiaaxMaacaWLjaWaaeWaaeaacqaI1aqnaiaawIcacaGLPaaaaaa@5284@

where *C *is a constant (see section 2 of [11]). One would expect that we can sharpen (5) by considering the probabilistic behavior of

Qmax⁡(n)def¯¯max⁡1≤i≤n{|hi−h˜i|}     (6)
MathType@MTEF@5@5@+=feaafiart1ev1aaatCvAUfKttLearuWrP9MDH5MBPbIqV92AaeXatLxBI9gBaebbnrfifHhDYfgasaacH8akY=wiFfYdH8Gipec8Eeeu0xXdbba9frFj0=OqFfea0dXdd9vqai=hGuQ8kuc9pgc9s8qqaq=dirpe0xb9q8qiLsFr0=vr0=vr0dc8meaabaqaciaacaGaaeqabaqabeGadaaakeaacqWGrbqudaWgaaWcbaGagiyBa0MaeiyyaeMaeiiEaGhabeaakiabcIcaOiabd6gaUjabcMcaPmaameaabaGaeeizaqMaeeyzauMaeeOzaygaamaaxababaGagiyBa0MaeiyyaeMaeiiEaGhaleaacqaIXaqmcqGHKjYOcqWGPbqAcqGHKjYOcqWGUbGBaeqaaOWaaiWabeaacqGG8baFcqWGObaAdaWgaaWcbaGaemyAaKgabeaakiabgkHiTiqbdIgaOzaaiaWaaSbaaSqaaiabdMgaPbqabaGccqGG8baFaiaawUhacaGL9baacaWLjaGaaCzcamaabmaabaGaeGOnaydacaGLOaGaayzkaaaaaa@549A@

For the moment, suppose that we can prove that Qmax⁡(n)≤C⋅(log⁡2nn)2α1+2α
 MathType@MTEF@5@5@+=feaafiart1ev1aaatCvAUfKttLearuWrP9MDH5MBPbIqV92AaeXatLxBI9gBaebbnrfifHhDYfgasaacH8akY=wiFfYdH8Gipec8Eeeu0xXdbba9frFj0=OqFfea0dXdd9vqai=hGuQ8kuc9pgc9s8qqaq=dirpe0xb9q8qiLsFr0=vr0=vr0dc8meaabaqaciaacaGaaeqabaqabeGadaaakeaacqWGrbqudaWgaaWcbaGagiyBa0MaeiyyaeMaeiiEaGhabeaakiabcIcaOiabd6gaUjabcMcaPiabgsMiJkabdoeadjabgwSixpaabmaabaWaaSaaaeaacyGGSbaBcqGGVbWBcqGGNbWzdaWgaaWcbaGaeGOmaidabeaakiabd6gaUbqaaiabd6gaUbaaaiaawIcacaGLPaaadaahaaWcbeqaamaalaaabaGaeGOmaidcciGae8xSdegabaGaeGymaeJaey4kaSIaeGOmaiJae8xSdegaaaaaaaa@4B34@ almost surely. This will not violate (5); but will strengthen it so that we can use (6) in the analysis of SR. However, there is a technical flaw in (6), which is an issue commonly overlooked in the current analysis on SR.

First, (5) and (6) can be used in signal processing since noise is always considered as a random variable with zero mean. However, in a mammalian auditory system, noise is a random variable with positive mean. For a mammalian auditory system, the baseline can logically be set at absolute silence and mathematically fixed at 0. When noise is used to sample an original signal, then it must be measured above the baseline, and hence, must have a positive mean. Accordingly, noise with a positive mean was used in [9]. Thus, we would expect h˜
 MathType@MTEF@5@5@+=feaafiart1ev1aaatCvAUfKttLearuWrP9MDH5MBPbIqV92AaeXatLxBI9gBaebbnrfifHhDYfgasaacH8akY=wiFfYdH8Gipec8Eeeu0xXdbba9frFj0=OqFfea0dXdd9vqai=hGuQ8kuc9pgc9s8qqaq=dirpe0xb9q8qiLsFr0=vr0=vr0dc8meaabaqaciaacaGaaeqabaqabeGadaaakeaacuWGObaAgaacaaaa@2E14@_*i *_>*h*_*i *_which in mathematical terms can be expressed as

h˜
 MathType@MTEF@5@5@+=feaafiart1ev1aaatCvAUfKttLearuWrP9MDH5MBPbIqV92AaeXatLxBI9gBaebbnrfifHhDYfgasaacH8akY=wiFfYdH8Gipec8Eeeu0xXdbba9frFj0=OqFfea0dXdd9vqai=hGuQ8kuc9pgc9s8qqaq=dirpe0xb9q8qiLsFr0=vr0=vr0dc8meaabaqaciaacaGaaeqabaqabeGadaaakeaacuWGObaAgaacaaaa@2E14@_*i *_= *h*_*i *_+ W
 MathType@MTEF@5@5@+=feaafiart1ev1aaatCvAUfKttLearuWrP9MDH5MBPbIqV92AaeXatLxBI9gBamrtHrhAL1wy0L2yHvtyaeHbnfgDOvwBHrxAJfwnaebbnrfifHhDYfgasaacH8akY=wiFfYdH8Gipec8Eeeu0xXdbba9frFj0=OqFfea0dXdd9vqai=hGuQ8kuc9pgc9s8qqaq=dirpe0xb9q8qiLsFr0=vr0=vr0dc8meaabaqaciaacaGaaeqabaWaaeGaeaaakeaaimaacqWFwe=vaaa@384D@_*i*_

Obviously, W
 MathType@MTEF@5@5@+=feaafiart1ev1aaatCvAUfKttLearuWrP9MDH5MBPbIqV92AaeXatLxBI9gBamrtHrhAL1wy0L2yHvtyaeHbnfgDOvwBHrxAJfwnaebbnrfifHhDYfgasaacH8akY=wiFfYdH8Gipec8Eeeu0xXdbba9frFj0=OqFfea0dXdd9vqai=hGuQ8kuc9pgc9s8qqaq=dirpe0xb9q8qiLsFr0=vr0=vr0dc8meaabaqaciaacaGaaeqabaWaaeGaeaaakeaaimaacqWFwe=vaaa@384D@_*i *_must be a constant; for otherwise, h˜
 MathType@MTEF@5@5@+=feaafiart1ev1aaatCvAUfKttLearuWrP9MDH5MBPbIqV92AaeXatLxBI9gBaebbnrfifHhDYfgasaacH8akY=wiFfYdH8Gipec8Eeeu0xXdbba9frFj0=OqFfea0dXdd9vqai=hGuQ8kuc9pgc9s8qqaq=dirpe0xb9q8qiLsFr0=vr0=vr0dc8meaabaqaciaacaGaaeqabaqabeGadaaakeaacuWGObaAgaacaaaa@2E14@_*i*_, the recovery from the noisy samples, will be skewed, which may cause a severe loss of information carried by *h*_*i *_(the original signal). On the other hand, W
 MathType@MTEF@5@5@+=feaafiart1ev1aaatCvAUfKttLearuWrP9MDH5MBPbIqV92AaeXatLxBI9gBamrtHrhAL1wy0L2yHvtyaeHbnfgDOvwBHrxAJfwnaebbnrfifHhDYfgasaacH8akY=wiFfYdH8Gipec8Eeeu0xXdbba9frFj0=OqFfea0dXdd9vqai=hGuQ8kuc9pgc9s8qqaq=dirpe0xb9q8qiLsFr0=vr0=vr0dc8meaabaqaciaacaGaaeqabaWaaeGaeaaakeaaimaacqWFwe=vaaa@384D@_*i *_are from the noise. Recall that we assumed that the noise is represented by a bounded random variable *e*(*t*) with 0 ≤ *a *≤ *e*(*t*) ≤ *b *where *a *<*b *are constants. Without loss of generality, we let the mean of this random variable be *m *= a+b2
 MathType@MTEF@5@5@+=feaafiart1ev1aaatCvAUfKttLearuWrP9MDH5MBPbIqV92AaeXatLxBI9gBaebbnrfifHhDYfgasaacH8akY=wiFfYdH8Gipec8Eeeu0xXdbba9frFj0=OqFfea0dXdd9vqai=hGuQ8kuc9pgc9s8qqaq=dirpe0xb9q8qiLsFr0=vr0=vr0dc8meaabaqaciaacaGaaeqabaqabeGadaaakeaadaWcaaqaaiabdggaHjabgUcaRiabdkgaIbqaaiabikdaYaaaaaa@3128@ > 0. The best scenario that one can expect is W
 MathType@MTEF@5@5@+=feaafiart1ev1aaatCvAUfKttLearuWrP9MDH5MBPbIqV92AaeXatLxBI9gBamrtHrhAL1wy0L2yHvtyaeHbnfgDOvwBHrxAJfwnaebbnrfifHhDYfgasaacH8akY=wiFfYdH8Gipec8Eeeu0xXdbba9frFj0=OqFfea0dXdd9vqai=hGuQ8kuc9pgc9s8qqaq=dirpe0xb9q8qiLsFr0=vr0=vr0dc8meaabaqaciaacaGaaeqabaWaaeGaeaaakeaaimaacqWFwe=vaaa@384D@_*i *_= *m *almost surely. For the moment, suppose this can be proven. Then, we can rewrite (6) as

Qmax⁡˜(n)def¯¯max⁡1≤i≤n{|(hi+m)−h˜i|}
MathType@MTEF@5@5@+=feaafiart1ev1aaatCvAUfKttLearuWrP9MDH5MBPbIqV92AaeXatLxBI9gBaebbnrfifHhDYfgasaacH8akY=wiFfYdH8Gipec8Eeeu0xXdbba9frFj0=OqFfea0dXdd9vqai=hGuQ8kuc9pgc9s8qqaq=dirpe0xb9q8qiLsFr0=vr0=vr0dc8meaabaqaciaacaGaaeqabaqabeGadaaakeaadaaiaaqaaiabdgfarnaaBaaaleaacyGGTbqBcqGGHbqycqGG4baEaeqaaaGccaGLdmaacqGGOaakcqWGUbGBcqGGPaqkdaadbaqaaiabbsgaKjabbwgaLjabbAgaMbaadaWfqaqaaiGbc2gaTjabcggaHjabcIha4bWcbaGaeGymaeJaeyizImQaemyAaKMaeyizImQaemOBa4gabeaakmaacmqabaGaeiiFaWNaeiikaGIaemiAaG2aaSbaaSqaaiabdMgaPbqabaGccqGHRaWkcqWGTbqBcqGGPaqkcqGHsislcuWGObaAgaacamaaBaaaleaacqWGPbqAaeqaaOGaeiiFaWhacaGL7bGaayzFaaaaaa@558C@

Hong and Birget [13] showed that with the threshold *λ*_*n,δ *_by Steps 1, 2 and 3 we have, for all *n *≥ 512

Pr⁡{Qmax⁡˜(n)≤(c1+c2δ)(log⁡2nn)α1+2α}≥1−9n1+δ     (7)
 MathType@MTEF@5@5@+=feaafiart1ev1aaatCvAUfKttLearuWrP9MDH5MBPbIqV92AaeXatLxBI9gBaebbnrfifHhDYfgasaacH8akY=wiFfYdH8Gipec8Eeeu0xXdbba9frFj0=OqFfea0dXdd9vqai=hGuQ8kuc9pgc9s8qqaq=dirpe0xb9q8qiLsFr0=vr0=vr0dc8meaabaqaciaacaGaaeqabaqabeGadaaakeaacyGGqbaucqGGYbGCdaGadeqaamaaGaaabaGaemyuae1aaSbaaSqaaiGbc2gaTjabcggaHjabcIha4bqabaaakiaawoWaaiabcIcaOiabd6gaUjabcMcaPiabgsMiJkabcIcaOiabdogaJnaaBaaaleaacqaIXaqmaeqaaOGaey4kaSIaem4yam2aaSbaaSqaaiabikdaYaqabaacciGccqWF0oazcqGGPaqkdaqadaqaamaalaaabaGagiiBaWMaei4Ba8Maei4zaC2aaSbaaSqaaiabikdaYaqabaGccqWGUbGBaeaacqWGUbGBaaaacaGLOaGaayzkaaWaaWbaaSqabeaadaWcaaqaaiab=f7aHbqaaiabigdaXiabgUcaRiabikdaYiab=f7aHbaaaaaakiaawUhacaGL9baacqGHLjYScqaIXaqmcqGHsisldaWcaaqaaiabiMda5aqaaiabd6gaUnaaCaaaleqabaGaeGymaeJaey4kaSIae8hTdqgaaaaakiaaxMaacaWLjaWaaeWaaeaacqaI3aWnaiaawIcacaGLPaaaaaa@6327@

where *c*_1 _and *c*_2 _depend only on (*b *- *a*), *M*, and *α*. (We note the following. In [13] the mean *m *of the random variable *e*(*t*) was supposed to be zero; however, with a trivial modification, all proofs can be applied when *m *> 0.) Since *n *is large and *δ *> 0, we have 1−9n1+δ
 MathType@MTEF@5@5@+=feaafiart1ev1aaatCvAUfKttLearuWrP9MDH5MBPbIqV92AaeXatLxBI9gBaebbnrfifHhDYfgasaacH8akY=wiFfYdH8Gipec8Eeeu0xXdbba9frFj0=OqFfea0dXdd9vqai=hGuQ8kuc9pgc9s8qqaq=dirpe0xb9q8qiLsFr0=vr0=vr0dc8meaabaqaciaacaGaaeqabaqabeGadaaakeaacqaIXaqmcqGHsisldaWcaaqaaiabiMda5aqaaiabd6gaUnaaCaaaleqabaGaeGymaeJaey4kaSccciGae8hTdqgaaaaaaaa@34A9@ and (log⁡2nn)α1+2α
 MathType@MTEF@5@5@+=feaafiart1ev1aaatCvAUfKttLearuWrP9MDH5MBPbIqV92AaeXatLxBI9gBaebbnrfifHhDYfgasaacH8akY=wiFfYdH8Gipec8Eeeu0xXdbba9frFj0=OqFfea0dXdd9vqai=hGuQ8kuc9pgc9s8qqaq=dirpe0xb9q8qiLsFr0=vr0=vr0dc8meaabaqaciaacaGaaeqabaqabeGadaaakeaadaqadaqaamaalaaabaGagiiBaWMaei4Ba8Maei4zaC2aaSbaaSqaaiabikdaYaqabaGccqWGUbGBaeaacqWGUbGBaaaacaGLOaGaayzkaaWaaWbaaSqabeaadaWcaaqaaGGaciab=f7aHbqaaiabigdaXiabgUcaRiabikdaYiab=f7aHbaaaaaaaa@3C96@ which are extremely close to 1 and 0, respectively. Thus, (7) indicates that the error Qmax⁡˜(n)
 MathType@MTEF@5@5@+=feaafiart1ev1aaatCvAUfKttLearuWrP9MDH5MBPbIqV92AaeXatLxBI9gBaebbnrfifHhDYfgasaacH8akY=wiFfYdH8Gipec8Eeeu0xXdbba9frFj0=OqFfea0dXdd9vqai=hGuQ8kuc9pgc9s8qqaq=dirpe0xb9q8qiLsFr0=vr0=vr0dc8meaabaqaciaacaGaaeqabaqabeGadaaakeaadaaiaaqaaiabdgfarnaaBaaaleaacyGGTbqBcqGGHbqycqGG4baEaeqaaaGccaGLdmaacqGGOaakcqWGUbGBcqGGPaqkaaa@360C@ is almost surely close to 0. A key step in proving (7) was to apply a deep result in measure concentration [15], a recently developed field in probability.

Summarizing all discussed thus far in this subsection, with the proposed model we can conclude that a mammalian auditory system processes an original subthreshold signal *h*(*t*) <*s *as follows. At time instants *t *= in
 MathType@MTEF@5@5@+=feaafiart1ev1aaatCvAUfKttLearuWrP9MDH5MBPbIqV92AaeXatLxBI9gBaebbnrfifHhDYfgasaacH8akY=wiFfYdH8Gipec8Eeeu0xXdbba9frFj0=OqFfea0dXdd9vqai=hGuQ8kuc9pgc9s8qqaq=dirpe0xb9q8qiLsFr0=vr0=vr0dc8meaabaqaciaacaGaaeqabaqabeGadaaakeaadaWcaaqaaiabdMgaPbqaaiabd6gaUbaaaaa@2F7C@, *i *= 1,2, ..., *n*, the intrinsic noise *e*(*t*) with mean *m *> 0 is employed to sample the original signal, generating the detectable noisy samples *f*(in
 MathType@MTEF@5@5@+=feaafiart1ev1aaatCvAUfKttLearuWrP9MDH5MBPbIqV92AaeXatLxBI9gBaebbnrfifHhDYfgasaacH8akY=wiFfYdH8Gipec8Eeeu0xXdbba9frFj0=OqFfea0dXdd9vqai=hGuQ8kuc9pgc9s8qqaq=dirpe0xb9q8qiLsFr0=vr0=vr0dc8meaabaqaciaacaGaaeqabaqabeGadaaakeaadaWcaaqaaiabdMgaPbqaaiabd6gaUbaaaaa@2F7C@) = *h*(in
 MathType@MTEF@5@5@+=feaafiart1ev1aaatCvAUfKttLearuWrP9MDH5MBPbIqV92AaeXatLxBI9gBaebbnrfifHhDYfgasaacH8akY=wiFfYdH8Gipec8Eeeu0xXdbba9frFj0=OqFfea0dXdd9vqai=hGuQ8kuc9pgc9s8qqaq=dirpe0xb9q8qiLsFr0=vr0=vr0dc8meaabaqaciaacaGaaeqabaqabeGadaaakeaadaWcaaqaaiabdMgaPbqaaiabd6gaUbaaaaa@2F7C@) + *e*(in
 MathType@MTEF@5@5@+=feaafiart1ev1aaatCvAUfKttLearuWrP9MDH5MBPbIqV92AaeXatLxBI9gBaebbnrfifHhDYfgasaacH8akY=wiFfYdH8Gipec8Eeeu0xXdbba9frFj0=OqFfea0dXdd9vqai=hGuQ8kuc9pgc9s8qqaq=dirpe0xb9q8qiLsFr0=vr0=vr0dc8meaabaqaciaacaGaaeqabaqabeGadaaakeaadaWcaaqaaiabdMgaPbqaaiabd6gaUbaaaaa@2F7C@) ≥ *s*. Then, by the Step 1, 2 and 3, the system recovers the noisy samples, obtaining h˜
 MathType@MTEF@5@5@+=feaafiart1ev1aaatCvAUfKttLearuWrP9MDH5MBPbIqV92AaeXatLxBI9gBaebbnrfifHhDYfgasaacH8akY=wiFfYdH8Gipec8Eeeu0xXdbba9frFj0=OqFfea0dXdd9vqai=hGuQ8kuc9pgc9s8qqaq=dirpe0xb9q8qiLsFr0=vr0=vr0dc8meaabaqaciaacaGaaeqabaqabeGadaaakeaacuWGObaAgaacaaaa@2E14@(in
 MathType@MTEF@5@5@+=feaafiart1ev1aaatCvAUfKttLearuWrP9MDH5MBPbIqV92AaeXatLxBI9gBaebbnrfifHhDYfgasaacH8akY=wiFfYdH8Gipec8Eeeu0xXdbba9frFj0=OqFfea0dXdd9vqai=hGuQ8kuc9pgc9s8qqaq=dirpe0xb9q8qiLsFr0=vr0=vr0dc8meaabaqaciaacaGaaeqabaqabeGadaaakeaadaWcaaqaaiabdMgaPbqaaiabd6gaUbaaaaa@2F7C@). (7) indicates that almost surely

|(h(in)+m)−h˜(in)|≈0for all i=1,2,...,n     (8)
 MathType@MTEF@5@5@+=feaafiart1ev1aaatCvAUfKttLearuWrP9MDH5MBPbIqV92AaeXatLxBI9gBaebbnrfifHhDYfgasaacH8akY=wiFfYdH8Gipec8Eeeu0xXdbba9frFj0=OqFfea0dXdd9vqai=hGuQ8kuc9pgc9s8qqaq=dirpe0xb9q8qiLsFr0=vr0=vr0dc8meaabaqaciaacaGaaeqabaqabeGadaaakeaafaqabeqacaaabaWaaqWaaeaadaqadaqaaiabdIgaOjabcIcaOmaalaaabaGaemyAaKgabaGaemOBa4gaaiabcMcaPiabgUcaRiabd2gaTbGaayjkaiaawMcaaiabgkHiTiqbdIgaOzaaiaGaeiikaGYaaSaaaeaacqWGPbqAaeaacqWGUbGBaaGaeiykaKcacaGLhWUaayjcSdGaeyisISRaeGimaadabaGaeeOzayMaee4Ba8MaeeOCaiNaeeiiaaIaeeyyaeMaeeiBaWMaeeiBaWMaeeiiaaIaemyAaKMaeyypa0JaeGymaeJaeiilaWIaeGOmaiJaeiilaWIaeiOla4IaeiOla4IaeiOla4IaeiilaWIaemOBa4gaaiaaxMaacaWLjaWaaeWaaeaacqaI4aaoaiaawIcacaGLPaaaaaa@5B71@

This means that the system amplifies the original subthreshold signal by simply translating it up with the mean *m *of the intrinsic noise. Figure 1 illustrates this. Some remarks need to made.

• Our analysis of SR takes an approach that differs substantially from the current view of SR as applied to sensory physiology. However, our approach does follow from the core idea by Moss [8] that noise enhances hearing by sampling the subthreshold signal. Using recent deep results in signal processing ([15,13]), our analysis further provides a strong statement that **a necessary and sufficient condition for SR to occur in a mammalian auditory system is that all the samples by the noise are detectable**.

• Our model and analysis do not involve energy and information modulation (as was also apparent in Moss' original description of SR in sensory systems [8]). However, we formulate this idea in a rigorous and concrete way: **using noise to sample an original subthreshold signal, a mammalian auditory system processes the noisy samples to translate the original signal up (in amplitude) by the mean of the noise**.

• A new insight that our model and analysis adds to SR is as follows: **When a mammalian auditory system processes the noisy samples, it may deposit energy into the recovered signal, and this added energy is expended in the recovery process**. As a consequence, our result suggests that information modulation is not a likely mechanism for SR, as discussed below.

Recall that in the analysis of a mammalian auditory system above, all signals and noise are evaluated in terms of their pressure against a fixed baseline. Thus, we can compute energies of signals and noise. The energy carried by an original subthreshold signal is

Esignal=∫01h(t)2dt
 MathType@MTEF@5@5@+=feaafiart1ev1aaatCvAUfKttLearuWrP9MDH5MBPbIqV92AaeXatLxBI9gBaebbnrfifHhDYfgasaacH8akY=wiFfYdH8Gipec8Eeeu0xXdbba9frFj0=OqFfea0dXdd9vqai=hGuQ8kuc9pgc9s8qqaq=dirpe0xb9q8qiLsFr0=vr0=vr0dc8meaabaqaciaacaGaaeqabaqabeGadaaakeaaieqacqWFfbqrdaWgaaWcbaGaee4CamNaeeyAaKMaee4zaCMaeeOBa4MaeeyyaeMaeeiBaWgabeaakiabg2da9maapedabaGaemiAaGMaeiikaGIaemiDaqNaeiykaKYaaWbaaSqabeaacqaIYaGmaaaabaGaeGimaadabaGaeGymaedaniabgUIiYdGccqWGKbazcqWG0baDaaa@438A@

Since the sample rate *n *is large, we can interpolate h˜
 MathType@MTEF@5@5@+=feaafiart1ev1aaatCvAUfKttLearuWrP9MDH5MBPbIqV92AaeXatLxBI9gBaebbnrfifHhDYfgasaacH8akY=wiFfYdH8Gipec8Eeeu0xXdbba9frFj0=OqFfea0dXdd9vqai=hGuQ8kuc9pgc9s8qqaq=dirpe0xb9q8qiLsFr0=vr0=vr0dc8meaabaqaciaacaGaaeqabaqabeGadaaakeaacuWGObaAgaacaaaa@2E14@_*i*_, *i *= 1, 2, ..., *n*, by segments to have a function h˜
 MathType@MTEF@5@5@+=feaafiart1ev1aaatCvAUfKttLearuWrP9MDH5MBPbIqV92AaeXatLxBI9gBaebbnrfifHhDYfgasaacH8akY=wiFfYdH8Gipec8Eeeu0xXdbba9frFj0=OqFfea0dXdd9vqai=hGuQ8kuc9pgc9s8qqaq=dirpe0xb9q8qiLsFr0=vr0=vr0dc8meaabaqaciaacaGaaeqabaqabeGadaaakeaacuWGObaAgaacaaaa@2E14@(*t*), *t *∈ [0,1]. This is equivalent to taking the Haar wavelet as the basis, and thus, will not violate our analysis presented above. Our analysis above showed h˜
 MathType@MTEF@5@5@+=feaafiart1ev1aaatCvAUfKttLearuWrP9MDH5MBPbIqV92AaeXatLxBI9gBaebbnrfifHhDYfgasaacH8akY=wiFfYdH8Gipec8Eeeu0xXdbba9frFj0=OqFfea0dXdd9vqai=hGuQ8kuc9pgc9s8qqaq=dirpe0xb9q8qiLsFr0=vr0=vr0dc8meaabaqaciaacaGaaeqabaqabeGadaaakeaacuWGObaAgaacaaaa@2E14@(*t*) ≡ *h*(*t*) + *m *almost surely. Hence, we can write the energy carried by the recovered signal as

Erecovery=∫01h˜(t)2dt=∫01(h(t)+m)2dt
 MathType@MTEF@5@5@+=feaafiart1ev1aaatCvAUfKttLearuWrP9MDH5MBPbIqV92AaeXatLxBI9gBaebbnrfifHhDYfgasaacH8akY=wiFfYdH8Gipec8Eeeu0xXdbba9frFj0=OqFfea0dXdd9vqai=hGuQ8kuc9pgc9s8qqaq=dirpe0xb9q8qiLsFr0=vr0=vr0dc8meaabaqaciaacaGaaeqabaqabeGadaaakeaaieqacqWFfbqrdaWgaaWcbaGaeeOCaiNaeeyzauMaee4yamMaee4Ba8MaeeODayNaeeyzauMaeeOCaiNaeeyEaKhabeaakiabg2da9maapedabaGafmiAaGMbaGaacqGGOaakcqWG0baDcqGGPaqkdaahaaWcbeqaaiabikdaYaaaaeaacqaIWaamaeaacqaIXaqma0Gaey4kIipakiabdsgaKjabdsha0jabg2da9maapedabaGaeiikaGIaemiAaGMaeiikaGIaemiDaqNaeiykaKIaey4kaSIaemyBa0MaeiykaKYaaWbaaSqabeaacqaIYaGmaaaabaGaeGimaadabaGaeGymaedaniabgUIiYdGccqWGKbazcqWG0baDaaa@57E9@

and hence,

Erecovery=∫01h(t)2+2m∫01h(t)dt+m2=Esignal+2m∫01h(t)dt+m2
 MathType@MTEF@5@5@+=feaafiart1ev1aaatCvAUfKttLearuWrP9MDH5MBPbIqV92AaeXatLxBI9gBaebbnrfifHhDYfgasaacH8akY=wiFfYdH8Gipec8Eeeu0xXdbba9frFj0=OqFfea0dXdd9vqai=hGuQ8kuc9pgc9s8qqaq=dirpe0xb9q8qiLsFr0=vr0=vr0dc8meaabaqaciaacaGaaeqabaqabeGadaaakeaafaqadeGabaaabaacbeGae8xrau0aaSbaaSqaaiabbkhaYjabbwgaLjabbogaJjabb+gaVjabbAha2jabbwgaLjabbkhaYjabbMha5bqabaGccqGH9aqpdaWdXaqaaiabdIgaOjabcIcaOiabdsha0jabcMcaPmaaCaaaleqabaGaeGOmaidaaaqaaiabicdaWaqaaiabigdaXaqdcqGHRiI8aOGaey4kaSIaeGOmaiJaemyBa02aa8qmaeaacqWGObaAcqGGOaakcqWG0baDcqGGPaqkaSqaaiabicdaWaqaaiabigdaXaqdcqGHRiI8aOGaemizaqMaemiDaqNaey4kaSIaemyBa02aaWbaaSqabeaacqaIYaGmaaaakeaacqGH9aqpcqWFfbqrdaWgaaWcbaGaee4CamNaeeyAaKMaee4zaCMaeeOBa4MaeeyyaeMaeeiBaWgabeaakiabgUcaRiabikdaYiabd2gaTnaapedabaGaemiAaGMaeiikaGIaemiDaqNaeiykaKcaleaacqaIWaamaeaacqaIXaqma0Gaey4kIipakiabdsgaKjabdsha0jabgUcaRiabd2gaTnaaCaaaleqabaGaeGOmaidaaaaaaaa@7216@

Recall that the energy carried by a random variable equals the deviation of the random variable. Thus, we can see that the energy carried by the intrinsic noise as a random variable is

**E**_noise _= *λm*^2 ^for 0 <*λ *< 1

where for a given noise *λ *is a constant. Therefore,

Erecovery=[Esignal+Enoise]=2m∫01h(t)dt+(1−λ)m2>0     (9)
 MathType@MTEF@5@5@+=feaafiart1ev1aaatCvAUfKttLearuWrP9MDH5MBPbIqV92AaeXatLxBI9gBaebbnrfifHhDYfgasaacH8akY=wiFfYdH8Gipec8Eeeu0xXdbba9frFj0=OqFfea0dXdd9vqai=hGuQ8kuc9pgc9s8qqaq=dirpe0xb9q8qiLsFr0=vr0=vr0dc8meaabaqaciaacaGaaeqabaqabeGadaaakeaaieqacqWFfbqrdaWgaaWcbaGaeeOCaiNaeeyzauMaee4yamMaee4Ba8MaeeODayNaeeyzauMaeeOCaiNaeeyEaKhabeaakiabg2da9iabcUfaBjab=veafnaaBaaaleaacqqGZbWCcqqGPbqAcqqGNbWzcqqGUbGBcqqGHbqycqqGSbaBaeqaaOGaey4kaSIae8xrau0aaSbaaSqaaiabb6gaUjabb+gaVjabbMgaPjabbohaZjabbwgaLbqabaGccqGGDbqxcqGH9aqpcqaIYaGmcqWGTbqBdaWdXaqaaiabdIgaOjabcIcaOiabdsha0jabcMcaPiabdsgaKjabdsha0bWcbaGaeGimaadabaGaeGymaedaniabgUIiYdGccqGHRaWkcqGGOaakcqaIXaqmcqGHsisliiGacqGF7oaBcqGGPaqkcqWGTbqBdaahaaWcbeqaaiabikdaYaaakiabg6da+iabicdaWiaaxMaacaWLjaWaaeWaaeaacqaI5aqoaiaawIcacaGLPaaaaaa@6C2C@

since *h*(*t*) > 0 and *m *> 0. In addition to the energies of the original signal and intrinsic noise, (9) indicates that as the noisy samples are processed, the auditory system itself adds an extra energy of amount 2m∫01h(t)dt+(1−λ)m2
 MathType@MTEF@5@5@+=feaafiart1ev1aaatCvAUfKttLearuWrP9MDH5MBPbIqV92AaeXatLxBI9gBaebbnrfifHhDYfgasaacH8akY=wiFfYdH8Gipec8Eeeu0xXdbba9frFj0=OqFfea0dXdd9vqai=hGuQ8kuc9pgc9s8qqaq=dirpe0xb9q8qiLsFr0=vr0=vr0dc8meaabaqaciaacaGaaeqabaqabeGadaaakeaacqaIYaGmcqWGTbqBdaWdXaqaaiabdIgaOjabcIcaOiabdsha0jabcMcaPaWcbaGaeGimaadabaGaeGymaedaniabgUIiYdGccqWGKbazcqWG0baDcqGHRaWkcqGGOaakcqaIXaqmcqGHsisliiGacqWF7oaBcqGGPaqkcqWGTbqBdaahaaWcbeqaaiabikdaYaaaaaa@42FE@ to the recovered signal. The extra energy allows SR to occur even if [**E**_signal _+ **E**_noise_] is not sufficient to reach threshold. Indeed, if one explained SR by energy addition, then it would be necessary that

[**E**_signal _+ **E**_noise_] ≥ *s*^2^

i.e., the added energy is at least more than a constant signal with intensity equal to the threshold *s*. Thus, when

[**E**_signal _+ **E**_noise_] <*s*^2 ^    (10)

then energy addition can no longer be used to explain SR. Moss and his coworkers called SR under condition (10) Type I, and asserted that it would require information modulation. However, an infinite number of signals *h *can be shown to satisfy the necessary and sufficient condition for SR to occur as suggested by our proposed model

*h*(*t*) + *m *≥ *s *all *t *∈ [0,1]

and yet have the property that

[**E**_single _+ **E**_noise_] <*s*^2^

Here, we present one example, as summarized in Figure 1. Suppose that the noise *e*(*t*) is characterized as 0.25 ≤ *e*(*t*) ≤ 0.32 and *m *= 0.285; and the threshold *s *= 1.0. Consider an original signal *h*(*t*) such that its intensity is within [0.75,0.85] and its average intensity is 0.8. Then the energy of *e*(*t*) is no more than 0.285^2 ^= 0.08, and the energy of *h *is no more than 0.8^2 ^= 0.64. Then 0.08 + 0.64 < 1 implies that the energy addition of *e*(*t*) and *h*(*t*) is not sufficient to explain how SR can enhance the reception of *h*(*t*). However, since *h*(*t*) + *e*(*t*) ≥ (0.75 + 0.25) = 1.0, the necessary and sufficient condition for our proposed model is satisfied and SR will occur (even without invoking information modulation).

### Physiological analysis

Our proposed model points out that the mammalian auditory system needs only to be capable of performing Steps 1, 2 and 3 to process a noisy signal. The mammalian auditory system has a long history of neuroanatomical, physiological and psychophysical analysis (cf. [16-23]) with which to draw parallels to the steps of this model. Moreover, SR phenomena have been clearly documented in this system [4,7], thus providing the impetus for modeling. Since our model was inspired by an analysis of SR in the mammalian auditory system, we find it important to consider how the steps of the processing might be performed. Since (1) indicates that noise is added directly to subthreshold signals, this process is likely to occur in the inner ear, the origin of the neural aspects of the auditory system. Processing of an auditory signal involves both transduction by hair cells and synaptic integration by innervating spiral ganglion neurons. Outer hair cells (OHC) insert energy into the signal as they modulate the stiffness of the tectorial membrane. Changes in stiffness of the tectorial membrane modulate transduction by the inner hair cells (IHC) and enhance signal transduction at near-threshold amplitudes. Evidence for this statement is implicit in the degradation of transduction capabilities by IHC's when OHC's are immobilized [24].

The IHC's drive spiking of spiral ganglion cells, but spiral ganglion axons also convey spikes in the absence of a signal [25]. Thus, the central output of the spiral ganglion appears to include added noise, as in (1). A spectrally complex signal is transduced by the spatially-organized frequency-based array formed at the cochlea and by the IHC's [23]. The spiral ganglion cells convey that information in their spike trains. These axons terminate in a spatially-organized pattern in the cochlear nuclei [23], thereby preserving the array derived at the cochlea.

The spiking activity within the orderly array of spiral ganglion cells and their central terminations in the cochlear nuclei can therefore be seen to represent the matrix of Step 1 and the thresholding operation of Step 2. Both frequency and amplitude information are simultaneously represented in the output of the array of spiral ganglion cells (eg, [23,26]). The orderly spatial mapping of the cochlea and cochlear nuclei is preserved in the serial pathway that includes the midbrain inferior collicular nucleus, the thalamic medial geniculate nucleus, and primary auditory cortex (e.g., [27-29]).

The neuroanatomical array maintains the signal representation to the cortex. Thus, the extensive representation of the cochlear array continued throughout the auditory system embodies the first two steps of our model.

It is currently difficult to precisely localize the anatomical site of occurrence of Step 3, the recovery of the signal. Step 3 is likely to occur sometime after primary auditory cortex, in which the array is also preserved [30-33]. Linguistic recognition in humans and animal recognition of species-specific vocalizations occurs beyond primary acoustic cortex [12,34], indicating that the reconstruction of a signal must also occur in higher order cortical areas involved in auditory function. Since the mammalian auditory system is capable of the concurrent recovery of frequency and amplitude information in short time segments [35,36], this, too, suggests a relationship between performance in the mammalian auditory system and our model based on wavelet analysis.

Hopfield [37,38] suggested that, as a consequence of evolution, interconnections built among a large number of simple neurons will form a stable network; and these networks compute [39]. Among the computational abilities of Hopfield networks are thresholding and linear transform (cf. [39]), both of which are required for our model. Hopfield networks may play the role of subsystems for DWT and its inverse. Thus, the mammalian auditory system, either at the stage of hair cell and spiral ganglion response integration (for Steps 1 and 2) or more centrally, in, for example, the auditory association cortex (for Step 3), may be considered as containing multiple Hopfield networks, and capable of the computations necessary for our model.

Central to our model's representation of SR is the realization that the system must add energy to the input (the initial signal and the noise) to exceed a perceptual threshold. To obey the first law of thermodynamics, the auditory system itself must therefore intrinsically add some energy to the noisy signal, and this extra energy is expended during the processing of information in Steps 1, 2 and 3. Three types of evidence suggest that this requirement is met experimentally. The first is the demonstration in mammalian hearing that a significant loss of threshold occurs with the loss of outer hair cell function [24]. Thus, one source of intrinsic energy might be embedded in the role of the OHCs. A second type of evidence is reflected in the physiology of eighth nerve afferents to the brain from the cochlea in the absence of a stimulus. Many studies of spiral ganglionic axons reveal classes of axons with different spontaneous activity (SA): one with Gaussian-like SA, one with bursting SA, and one with little SA. Spontaneous activity in an axon reflects an intrinsic property of the system that correlates with the sensitivity at an axon's characteristic frequency. Even in kittens raised in the absence of obvious sound stimuli, 8th nerve axons of these animals carry spontaneous activity [40]. Thirdly, in experiments with implanted cochlear electrodes in deaf people, Zeng et al. [9] showed that the addition of noise (i.e., extra energy) to a defined signal enhanced the perceptual sensitivity when near threshold levels.

In summary, the auditory system of mammals contains the necessary elements for using SR to process acoustic information according to the requirements and steps of our proposed model.

## Conclusion

We present a new theoretical viewpoint for the analysis of SR in the mammalian auditory system. Most strikingly, the analysis indicates that the mechanism for reception of auditory sensation is necessarily more active than previously considered.

Although energy-requiring aspects of cochlear function have been described previously [24], the current analysis indicates that the addition of energy is a key feature of auditory receptor function. The new model suggests that the effect of noise is to carry out a geometric translation, "lifting" the original signal by the mean of the noise and creating a noisy signal which is above threshold and discernable (see Figure 1). The result of this geometric translation is more than the energy addition of the original (subthreshold) signal and intrinsic noise.

The model shows that the mechanism underlying the geometric translation does not need to be very complex. The function of the mammalian auditory system can be modeled very simply in three steps by a DWT, followed by thresholding and the inverse of DWT. Wavelet analysis is considered a useful model of the auditory system because of the capability to concurrently represent temporal and intensity information in short time segments. Furthermore, the parameters used in the DWT, thresholding and inverse DWT are invariant and the processing can therefore proceed instantaneously. Since the parameters are invariant, they are components of the phenotype and therefore would be subject to natural selection. The mammalian auditory system, optimized by evolution, appears to have evolved unique specializations to take advantage of the phenomenon of SR to enhance sensory perception. The auditory system should be considered as an active, not passive, receptor.

## Authors' contributions

DH carried out the mathematical derivations and the drafting and review of the manuscript. JVM and WMS participated in the analysis of the model and the revision of the manuscript
